# A mixed-methods case study exploring the impact of participation in community activity groups for older adults on physical activity, health and wellbeing

**DOI:** 10.1186/s12877-019-1245-5

**Published:** 2019-09-02

**Authors:** Gabrielle Lindsay-Smith, Rochelle Eime, Grant O’Sullivan, Jack Harvey, Jannique G. Z. van Uffelen

**Affiliations:** 1 Institute for Health and Sport, Victoria University, Melbourne, Australia; 2 School of Health and Life Sciences, Federation University, Ballarat, Australia; 3 Department of Movement Sciences, Physical Activity, Sports and Health Research Group, KU Leuven - University of Leuven, B-3000 Leuven, Belgium

**Keywords:** Ageing, Longitudinal, Wellbeing, Health, Social-engagement

## Abstract

**Background:**

Regular physical activity (PA) has many health benefits but declines with age. Community multi-activity groups offering volunteer-led socially-oriented activity programs could provide an opportunity for older people to maintain or increase PA levels and promote their health. The aim of this study was to examine the potential effect of becoming a member of an existing community activity group on PA levels, physical and mental health-related quality of life (HR QoL), comparing any impacts associated with participation in physical activity or social activity programs.

**Methods:**

This mixed-methods case study, combining a longitudinal quantitative-survey with qualitative focus groups to contextualise the survey results, focused on an Australian community organisation called Life Activities Clubs (LACs). LACs provide various physical activities (e.g. walking, cycling, dancing) and social activities (e.g. book groups, dine-outs, craft). Data were collected using a self-report survey administered at baseline, six and twelve-months after joining and group differences between participants of PA programs (PA group) and social programs (social group) were analysed using linear mixed-models. Two focus groups with LAC members were held, one representing each activity type and analysed using content and thematic analysis.

**Results:**

35 people (mean age 67) completed the surveys and 11 people participated in the focus groups. PA levels and physical health-related QoL were maintained over 1 year in the PA group, and declined between baseline and 12-months in the social group. Focus groups suggested social aspects of PA programs increased motivation to maintain regular attendance and do more PA than participants would on their own and that physical activities provided health benefits. Mental HR QoL did not change in either group, focus groups suggested this was because the social aspects of both types of program provide benefits relating to mental health including stress relief, enjoyment and adapting to major life events, to prevent a decline in QoL.

**Conclusions:**

Community PA programs appear to maintain PA levels and physical HR QoL in older adults, and both social and PA programs may maintain mental HR QoL. Incorporating both types of program into one organisation may also encourage less physically active members to try physical activities.

## Background

Between 2015 and 2050 it is predicted that the number of people globally over the age of 60 will more than double [1]. Ageing is typically associated with increased risk of non-communicable diseases, functional decline and age-related conditions such as dementia, as well as a greater risk of being lonely or socially isolated [2–4]. This places significant burden on health and social care systems and can be detrimental to the quality of life of older adults themselves. It is therefore an individual and public health priority to focus on strategies that promote ‘Active Ageing’ (also sometimes referred to as healthy or successful ageing); defined as “the process of developing and maintaining the functional ability (physical and social characteristics) that enables well-being in older age” ([5], p28).

Physical activity is an essential component of such strategies because of its vastly known mental and physical health benefits such as chronic disease prevention [6–8], maintenance of functional capacity and cognitive health [9–13]. Despite the wide-ranging benefits of participating in regular PA, global inactivity levels are high and increase with age. Approximately 60–70% of older adults in developed countries such as Canada, the United States of America (USA) and Australia are not sufficiently active [14–17]. Based on this evidence, there is clearly a need for exploration of ways to improve PA levels of older adults that can be sustainable in the long-term. Older adults place greater importance on enjoyment and socialisation than their younger counterparts [18], and socially-oriented strategies are more effective for PA initiation and maintenance than purely individual strategies such as action planning, goal setting or barrier management in older adults [19–23]. Therefore, exploration of the potential of socially focused sustainable PA interventions for older adults is warranted.

One place where such strategies are integral to the service they offer are community organisations that run a variety of socially focused physical activity and social programs for older adults. For ease, these will be referred to as community activity groups. Such groups are generally sustainable, cost effective, often run by volunteers and developed by community members, making them an ideal setting for the promotion of Active Ageing [24–29]. These organisations offer people the opportunity to do the things they enjoy in a group environment, which can provide social wellbeing benefits such as social connection, reduced perception of loneliness and possibly increase social support [30]. Socialising in groups is also important for the cognitive and physical health and wellbeing of older adults [31–34]. In particular, older adults who participate in either sports or hobby groups have a lower risk of onset of functional disability and better QoL 4 years later compared to those who did not participate in any groups [35].

One potential mechanism is that group participation may strengthen social identification, leading to increased perceived social support [31, 36, 37]. Social support may buffer stressful situations [38–40] and/or encourage positive health behaviours, including PA [41–43]. Community based PA programs can increase PA levels in older adults [44], and appear to have good adherence of approximately 70% [45]. Some of the key factors that impact adherence to these types of programs are social connection, fun from socialising and social support from the group [45–47]. Community groups offering socially-focused PA may therefore have potential to increase PA levels and wellbeing for older adults.

Evaluation of community activity groups would help to identify strategies that promote healthy ageing and are sustainable in a real-life community-based setting. However, research in this setting is scarce, with most of the research in the field incorporating community PA groups as just one option in larger PA interventions in addition to individual strategies [48–51]. There is also a lack of longitudinal studies evaluating the impact of programs run in existing community organisations for older adults on PA and QoL. Given that community organisations are low cost and sustainable and offer physical activity programs with a socially oriented focus that some older adults prefer, the potential of these organisations warrants further investigation.

### Research objectives

The aim of this mixed-methods case study was to examine the potential effect of becoming a member of an existing community activity group on PA levels and QoL, and to compare the effects of participating in physical activity or social activity programs on these outcomes (quantitative research), and to explore this in depth with both new and longer-term members of the same community organisation (qualitative research).

## Methods

### Setting

#### Life activities clubs Victoria

Life Activities Clubs Victoria (LACVI) is a large not-for-profit community organisation with 23 independently run Life Activities Clubs (LACs) with approximately 4000 members based in both rural and metropolitan Victoria, Australia. The organisation was established in 1972 to provide physical, social and recreational activities, as well as education and motivational support, to older adults managing retirement and other significant changes in their lives. LACs offer a variety of types of activities depending on the individual club. Some examples of social activities include book groups, dine-outs, travel, craft or cultural activities. PA programs typically include walking, table-tennis, cycling or dancing [28]. Individuals can take part in unlimited activities for a small yearly fee membership fee of LACs,.Eighteen out of 23 LACs agreed to participate in the survey study.

### Participants

#### Survey

**Fig. 1:**
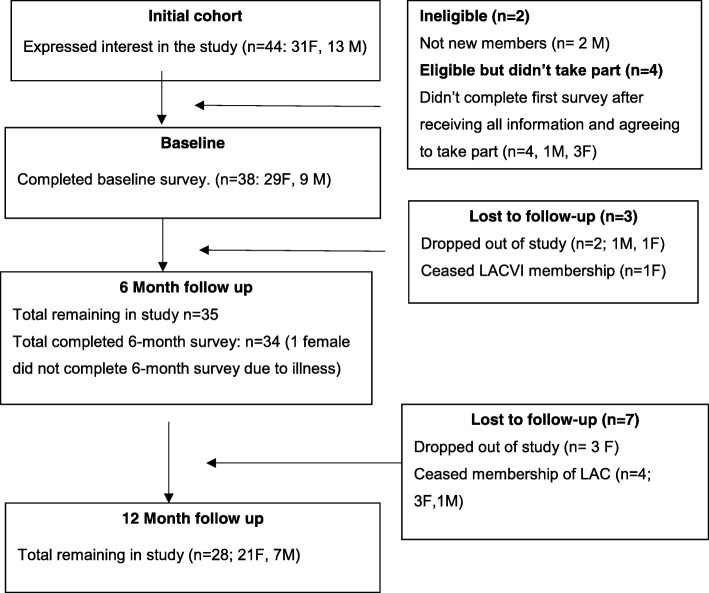
Participant recruitment flow chart

During the sampling period from May 2014 to December 2016, new members from participating LACs were given information about the survey study and invited to take part. Invitations in the form of flyers were included with new membership material. Eligibility criteria were as follows: 1) community-dwelling older adults who self-reported that they could walk at least 100 m; 2) new members of LACVI (defined as people who had never been members of LACVI or who had not been members in the last 2 years); 3) able to complete a survey in English. Thirty-five participants enrolled in the survey study (See Fig. 1 for full flow chart of survey participant recruitment). Due to the observational nature of the study, individuals self-selected their preferred programs rather than being randomly allocated. Seventeen participants chose to take part in social programs (social group) and 18 participants took part in PA programs (PA group).

#### Focus groups

Each of the survey participants were given an opportunity to participate in the focus groups (FGs). To gather additional views from longer-term members of the club, recruitment for the FGs was opened to all LACVI members and advertised through the LACVI newsletter. Eleven members participated in the FG study, seven of whom also completed the survey study. Two FGs were conducted to allow for comparison between groups; one containing social program participants (e.g. book groups, social groups, craft or cultural groups; *n* = 5) the other with PA program participants (e.g. walking groups, tennis, cycling; *n* = 6).

### Design

#### Survey

The survey was administered upon becoming a member and six and 12 months after joining. It was completed via self-report, either online or paper depending on participant preference. 13 participants (37%) completed the survey on paper and 22 were online (63%).

### Dependent variables

#### a) Physical activity (PA)

PA was assessed using the validated Active Australia Survey [52]. It assesses total minutes of PA undertaken in the previous week by summing bouts of 10 min of PA in each of the three categories (walking, moderate-intensity PA and vigorous-intensity PA) [53]. This measure has acceptable validity and reliability in adults and older adults [54, 55]. A total PA score in MET.hours/week was calculated by multiplying minutes in each activity type by an assigned metabolic equivalent (MET), summing and dividing by 60 (walking = 3.0 METs; moderate-intensity PA = 4.0 METs; vigorous-intensity PA = 7.5 METs) [56, 57]. PA was then truncated to a maximum of 112 MET.hours/week [56, 57]. PA was categorised as: 1) no PA (< 0.67 MET.hours/week); 2) insufficient PA (0.67 < 10 MET.hours/week); 3) sufficient PA (> 10–20 MET.hours/week) which was calculated as meeting WHO guidelines equivalent to 150 min/week or 2.5 h/week of moderate-intensity PA (2.5 h × 4 METS = 10 MET.hours) [8]; 4) double the recommended levels to gain health benefits [8] = 20 MET.hours/week) [58]. Missing data for this variable were not imputed. One participant (3%) had missing data on the AA questionnaire at 6 months.

#### b) Quality of life

Physical and mental health-related quality of life (HR QoL) was assessed using the Short form 12-item Health Survey Questionnaire version 2 (SF-12) [59]. The SF-12 consists of 12 questions relating to eight concepts of physical and mental health and how they impact one’s QoL, i.e., physical functioning, role-physical, bodily pain, general health, energy, social functioning, role emotional and mental health. The concepts are divided into two summary scores using norm-based criterion referred to as a physical component score (PCS) and mental component score (MCS), representing physical and mental HR QoL. The scores for each component are presented as standardised scores (M = 50, SD = 10). For example, a score of 60 represents a QoL rating one standard deviation higher than the average rating of the general population [60]. SF-12 has good internal consistency and test-retest reliability (for both alpha > 0.7), as well as good construct validity for use in older adults [61].

### Sociodemographic and health variables

The following sociodemographic characteristics were collected in both the survey and the FGs to describe the study sample: age, sex, highest level of education, main life occupation [62], current employment, ability to manage on income available, present marital status, country of birth, area of residence [63] (see Table 1). Self-rated general health was assessed with the question ‘In general, would you say your health is: excellent, very good, good, fair, poor?’ [60].

**Table 1:** Baseline Demographic and health characteristics of survey and focus group respondents *n* (%)

Sociodemographic characteristics		Survey (*n* = 35)			FGs (*n* = 11)
		Social (*n* = 17)	PA (*n* = 18)	Total	
Age in years, mean (SD)		67 (7)	67 (9)	67 (8)	67 (6)
Sex, *n* (%)	Male	2 (12)	6 (33)	8 (23)	2 (18)
	Female	15 (88)	12 (67)	27 (77)	9 (82)
Highest level of education, *n* (%)	Completed primary school			0 (0)	1 (9)
	Up to year 12	8 (47)	6 (33)	14 (40)	3 (27)
	Technical studies/ trade certificate	6 (35)	8 (44)	14 (40)	4 (36)
	Tertiary studies	3 (18)	4 (22)	7 (20)	3 (27)
Main life occupation, *n* (%)	Manager	4 (23.5)	2 (11)	6 (17)	2 (18)
	Professional	4 (23.5)	8 (44)	12 (34)	4 (36)
	Clerical	6 (35)	5 (28)	11 (31)	5 (45)
	Trade, production or labour	3 (18)	3 (17)	6 (17)	0
Current employment, *n* (%)	Full-time	1 (6)	2 (11)	3 (9)	0
	Part-time/casual	3 (18)	1 (6)	4 (11)	2 (18)
	Not in paid employment	13 (76)	15 (83)	28 (80)	9 (81)
Ability to manage on income, *n* (%)	Very difficult	1 (6)	1 (6)	2 (6)	–
	Somewhat difficult	6 (35)	3 (17)	9 (26)	3 (27)
	Not difficult	10 (59)	14 (78)	24 (68)	8 (18)
Present marital status, *n* (%)	Not married	11 (65)	8 (44)	19 (54)	8 (73)
	Married/de-facto	6 (35)	10 (56)	16 (46)	3 (27)
Country of birth, *n* (%)	Australia	17 (100)	10 (56)	27 (77)	8 (73)
	Other	0 (0)	8 (44)	8 (23)	3 (27)
Area of residence, *n* (%)	Urban	13 (76)	17 (94)	30 (86)	9 (82)
	Rural	4 (24)	1 (6)	5 (14)	2 (18)
General health, *n* (%)	Very good- excellent	11 (65)	11 (60)	22 (63)	
	Good	4 (23)	6 (33)	10 (28.5)	
	Fair	2 (12)	1 (5.5)	3 (8.5)	
PA levels *n* (%)	No PA	0	1 (6)	1 (3)	
	Insufficient PA	4 (24)	2 (11)	6 (17)	
	Sufficient PA	5 (29)	0	5 (14)	
	Enough PA for additional health benefits	8 (47)	15 (83)	23 (66)	

*SD* standard deviation

#### Focus groups

Qualitative data were collected in focus group discussions utilising a semi-structured interview format. The questions focused on the perceived health, wellbeing and PA benefits of being a member of a LAC and the perceived mechanisms for these benefits. This provided an opportunity for participants to disclose knowledge that was not otherwise captured through the survey alone. A semi-structured interview guide and the use of open-ended questions elicited broad discussion around health and wellbeing changes through program participation [64].

### Procedure

#### Survey

All participants provided written informed consent to participate in this study. See [30] for further details of data collection procedures for this study.

#### Focus groups

The FG interviews were facilitated by one researcher (GLS) and notes around non-verbal communication, moments of divergence and convergence amongst group members, and other notable items were taken by a second researcher (GOS). See [30] for further procedural details.

Ethics approval to conduct this study was obtained from the Victoria University Human Research Ethics Committee (HRE14–071 and HRE15–291) All participants provided written informed consent to partake in the study.

### Analysis

In line with recommendations, the synthesis of survey and FG data was undertaken during interpretation of the results [65].

#### Survey

Dependent variables (PA, SF-12 (MCS and PCS), were analysed in SPSS for windows (v25) using linear mixed models (LMM). LMM enables testing for the presence of intra-subject random effects, or equivalently, correlation of subjects’ measures over time (baseline, six-months and 12 months) and does not automatically remove cases from analysis if a single data point is missing. Three correlation structures were examined: independence (no correlation), compound symmetry (constant correlation of each subjects’ measures over the three time-points) and first-order autoregressive (AR1) (correlation diminishing with increase in spacing in time). The best fitting correlation structure for the three dependent variables was AR1. The LMMs incorporated terms for differences between the two groups (PA and social group), longitudinal trends over time and group-time interactions, with adjustment for age, employment and weekly frequency of attendance at the LAC program as potential confounders. Group by time interactions represent differences in the changes over time between the two groups. Residuals for PA were not normally distributed and the scores for these variables were therefore square-root transformed for statistical analysis and the median (Interquartile range = IQR: 25th–75th percentile) was reported in Table 2. An alpha level of 0.05 indicates statistical significance for main effects. A Bonferroni corrected alpha of 0.025 was utilised for post-hoc testing.

**Table 2:** Frequency of attendance at LAC in last month for social and PA groups indicated in the survey *n* (valid n%)

	Social group		PA group	
	6 months n(%)	12 months n(%)	6 months n(%)	12 months n(%)
Never	0	3 (21)	0	4 (29)
Infrequent (<1xpw)	7 (50)	5 (36)	9 (56)	4 (29)
Moderate (1-2xpw)	5 (36)	5 (36)	1 (6)	3 (21)
Frequent (>2xpw)	2 (14)	1 (7)	6 (38)	3 (21)

#### Focus groups

Focus group transcripts were analysed using a hybrid of descriptive content analysis [66] and thematic analysis [64, 67]. The transcribed data were analysed using a combination of deductive and inductive thematic analysis [67]. Deductive thematic analysis sought to assess the hypothesis that membership of a LAC would promote PA and QoL. Semantic themes were inductively drawn from these codes to conduct a pragmatic evaluation of the LAC programs [67]. Analytic rigour in the qualitative analysis was ensured through source and analyst triangulation [64]. Transcriptions were compared to notes taken during and immediately after the FGs by the researchers (GOS and GLS). In addition, initial coding and themes (by GLS) were checked by a second researcher (GOS) and any disagreements regarding coding and themes were discussed to find consensus on final codes and themes. Descriptive content analysis sought to describe the frequency of code and theme mentions. Frequency was determined by counting of mentions of each theme within the text. Counts were determined both by number of participants who mentioned a code (if more than one participant says the same thing), and by number of mentions by the same participant (if a participant says the same thing more than once). However, if the group indicated agreement with a point made by one participant by nodding or saying “Mmm” or “yeah” and thus, were not individually identified, this was not counted as extra mentions. The content analysis sought to identify the range and prominence of physical and psychological benefits of participation in LAC programs. The benefit themes and codes that were identified were then compared across the social and PA groups. Further exploration of thematic content was conducted once group differences had been identified. Group agreement with themes was considered at this point. See Table 3 for themes and numeric results of the content analysis and text below for detail of thematic analysis between individuals and groups in the study.

**Table 3:** Physical wellbeing variables over time in full group, and social and PA groups separated

	Group	B/L (*n* = 35)		6 month (*n* = 35)		12 month (*n* = 32)		Time effect	
		Med	IQR	Med	IQR	Med	IQR	F	P
PA	Total	33.3	12.2–55.4	22.6	10.5–50.3	32.5	7.5–42	1.34	0.270
	Social	15.0	11.5–36.8	10.5	7–50	7.5	3–39.8	2.04	0.140
	PA	42.0	25.5–65.3	36.2	21.5–56	38.5	13.5–51.5	0.12	0.884
	Group effect: F = 8.97; *p* = 0.005*				Group x time: F = 0.830; *p* = 0.441				
PCS^b^		M	SE	M	SE	M	SE	F	P
	Total	49.4	1.3	49.2	1.3	48.0	1.3	0.549	0.581
	Social	46.8	1.8	45.6	1.9	43.3	1.9	1.37	0.261
	PA	51.9	1.8	52.9	1.8	52.8	1.9	0.169	0.845
	Group effect: F = 13.1; *p* = 0.001*				Group x time: F = 0.999; *p* = 0.374				
MCS ^c^	Total	53.4	1.4	54.5	1.4	54.7	1.4	0.561	0.573
	Social	53.4	1.9	54.5	2.0	54.6	2.0	0.257	0.774
	PA	53.4	1.9	54.4	1.9	54.8	2.0	0.311	0.734
	Group effect: F = 0.001; *p* = 0.981				Group x time: F = 0.008; *p* = 0.992				

*B/L* Baseline, *IQR* inter quartile range (25-75th percentile), Med median (not adjusted), *M* Mean, *SE* standard error. * indicates a significant result for the corresponding variable. *p* < 0.05. All Analyses conducted using linear mixed model. AR1 correlation structure adjusted for age, employment, frequency of attendance. ^a^ *PA* MET.hours/week of PA. The residuals were skewed; *p*-value was calculated using a square-root transformation in the LMM. For readability, actual MET. Hour values are reported in this table not the transformed variable. ^**b**^*PCS* Physical component of the SF-12 (Mean and SE age adjusted). ^c^ *MCS* Mental component of the SF-12 (Mean and SE age adjusted)

## Results

### Survey

There were no significant differences between the sociodemographic characteristics of the participants of the PA group and social group; with a mean age of 67 (range 45–80) and 77% female. The demographic characteristics were also similar between the survey participants and the FG participants (see Table 1 for full details).

#### Frequency of attendance at LACVI and intensity of PA sessions at LAC

At six and 12 months, survey participants indicated how many times in the previous month they had attended activities at their LAC (see Table 4). Most participants maintained the same frequency of participation over both time points, although participation rates in some people declined. This was similar for both the Social and PA group (see Table 4). PA participants were asked to indicate the type and average intensity of PA done at their LAC. The main types of PA which participants were involved were walking, table tennis, bowls and dancing. At six-month and 12-month follow-up the majority of participants (78 and 88% respectively) rated the intensity of sessions they attended as moderate-vigorous and the rest rated it as low.

**Table 4:** Content analysis of mentions in each theme from the focus group study

Theme	Subtheme	PA ^a^	Social ^a^ a
Physical benefits	Improved physical capacity*	2	0
	General physical health*	5	0
Total		7	0
PA benefits	Decrease sitting time *	1	0
	New opportunities to do PA *	0	2
	More PA quantity/ intensity *	5	0
	PA Maintenance*	5	0
Total		18	2
Psychological benefits	Adapt to major life events	9	3
	Cognitive stimulation	4	2
	Improved mental health (general)*	5	0
	Improved life gratitude/ life satisfaction/ QoL	2	2
	Stress reduction/ relaxation	1	2
	Enjoyment	5	6
Total		26	15

^a^ These columns are counts of the number of mentions relating to each theme within each FG discussion. * Indicates that the subtheme was only mentioned in one group

#### Survey outcome measures

##### a) Physical activity

All participants had high initial levels of PA (see Table 1), with the majority (80%) undertaking sufficient amounts of PA and 66% doing more than double the recommended minimum to achieve health benefits. This was especially the case for the PA group, with 83% doing more than double the recommended minimum; a significantly greater proportion than the social group (47%) (χ^*2*^ = 0.024, DF = 1, *p* < 0.05)(see Table 3). There was a significant between-group effect of membership over time on PA (F [1, 32]=8.97, *p* = 0.005), with the PA group having significantly greater mean PA levels over time compared to the social group. The was no significant change in PA levels over time in either group (main effect of time time) and there were no significant differences between groups (group by time interaction)(see Table 2). However, participants in the focus groups felt that PA program participation was beneficial for their PA levels (see focus group results below). Therefore, post hoc analyses were conducted. There was no significant difference in PA between the groups at baseline or 6 months (F [1, 68]=3.37, *p* = 0.061), but the difference became significant by 12 months, with the PA group having an median total PA of 38.5 MET.hours/week of PA compared to 7.5MET.hours/week in the social group (F [1, 69]=9.29, *p* = 0.003). In addition, there was a trend toward a significant decline in PA levels in the social group between baseline and 12 months (*p* = 0.05 – not significant with a Bonferroni-adjusted *p*-value of 0.025) (see Table 3).

##### b) Quality of life

There was a significant difference between the mean physical HR QoL scores (PCS) of the participants in the two groups with the PA group having significantly higher mean PCS scores than the social group. Means and standard errors were 53.2 (2.06) for the PA group versus 44.8 (2.1) for the social group (F [1, 30]=13.1, *p* = 0.001)(see Table 3). The was no significant change in PCS scores over time in either group (main effect of time time) and there were no significant differences between groups (group by time interaction). However, the focus group discussion suggested that PA program participants felt they gained physical health benefits from group membership (see focus group results below), thus post-hoc analyses were conducted. The social group participants had significantly lower PCS scores than the PA group at six-months F [1, 52] = 9.36, *p* = 0.003 and 12-months F [1, 56]=13.75, *p* = 0.001) but not baseline and there was a trend toward significant decline in PCS scores in the social group between baseline and 12-months (*p* = 0.107)(see Table 3).

There were no significant differences in mental component of HR QoL over time (i.e. time effect), between groups (i.e. group effect) or differences in MCS over time between groups (group by time interaction).

### Focus groups

Six people (four women and two men) participated in the PA focus group and five (all women) participated in the social focus group (FG). FG participants were either retired (*n* = 9) or semi-retired (*n* = 2). The mean age of participants in the focus groups (FGs) was 67 years (range 55–78 years) (see Table 1 for further details). Most of the participants (82%) had been members of a LAC for less than 2 years and two women in the social group had been members of LACs for 5 and 10 years respectively. Analysis of the FG transcripts identified three themes relating to health benefits of participating in community activity group programs; 1) PA benefits, 2) physical benefits and 3) psychological benefits. In addition to benefits that were derived from both the PA and the social programs, several benefits were derived only from one type of program. These are summarised in Table discussed below.

#### PA benefits of participation in community activity group programs

The transcripts were coded for any discussion relating group program participation to PA levels. Analysis of the codes showed themes relating to impacts of program participation on PA (*physical activity benefits*) and also some discussion of mechanisms relating PA program involvement to the PA benefits (*physical activity mechanisms*). Within the PA benefits theme, four subthemes were identified, *i) Maintain PA*, *ii) More PA quantity/intensity iii) Decrease sitting time* and iv) *new opportunities to do PA*. Three of these (*i to iii*) related to involvement in PA programs only and further coding revealed four potential mechanisms linking involvement to PA levels and are detailed below. The final benefit of participation in community activity groups related potential opportunities for PA derived from social programs and was coded as *iv) new opportunities to do PA,* related to social program involvement only (see below and Table 3 for content analysis results).

### PA programs

#### Physical activity benefits

*i) Maintain PA:* This was the primary theme of this subsection. All the participants interviewed in the PA group agreed that the group assisted them in maintaining regular physical activity*.* For example, when asked about benefits of joining LACs, one man said, and others agreed that *“for me it’s mainly health benefits, more activity”.*

*ii) More PA quantity/intensity*: Approximately half the participants felt that participating in group PA motivated them to do a greater volume or intensity of PA than they would if they were exercising alone. This is illustrated in the following comment by one of the male participants: *“I wouldn’t be exercising as much as what I do now if it wasn’t for LAC”.*

*iii) Decrease sitting time* was mentioned as a benefit relating to group membership by one male participant who felt that preparation relating to his chosen activity made him less sedentary in his normal life outside the group: *“every week you’ve gotta prepare. … the [equipment] and make sure that it’s right to go, and that’s a matter of just doing something different, you know, you’re not sitting on your backside at home.*”

#### Potential mechanisms for increasing physical activity in PA programs

**Fig. 2:**
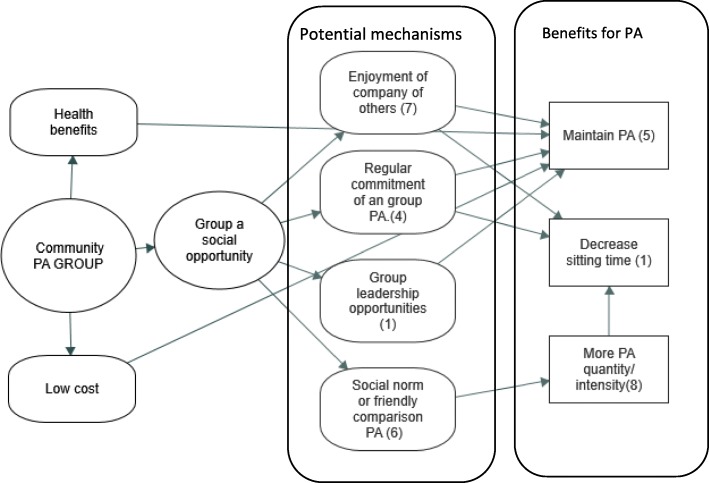
Potential mechanisms linking participation in a PA program and changes in PA

Four subthemes identified in the PA focus group coding related to participant perceptions of *how* participation in the PA programs influenced their PA levels. These were *i) enjoyment of the company of others ii) leadership opportunities through group membership iii) regular commitment of an activity and iv) social norm or friendly comparison of PA.* Figure 2 shows how each of the subthemes related to the type of PA benefit. The first three subthemes appeared to be related to decreased sitting time or maintenance of PA and the last subtheme specifically related to increased PA quantity.

i*) Enjoyment of the company of others* was the most mentioned subtheme in this section, with all members in the FG mentioning that they enjoyed socialising in their PA group and this motivated their continued attendance. One woman in the group made a pertinent point summarising the feeling of the group: *“Yeah, if, if you’re enjoying, say, something like table tennis or, or you’re bike riding or anything like that or dancing, I mean, if you’re enjoying the company of the people … that you’re sharing the activity with, I think it’s enough to sort of make you keep, keep going”.*

*ii) Leadership opportunities through group membership:* One male in the group mentioned that there was potential for leadership activities; to lead the rest of the group in the activity for the week, which he found motivating for his PA levels and attendance *“You are a leader for probably two (sessions) a year so you’ve gotta prepare for it, so that’s something that’s a responsibility”*.

*iii) Regular commitment of an activity:* Approximately half the group stated they found a regular group commitment of PA very motivating for continuing to exercise, especially when they had a set-back such as an injury or holiday. For example, one woman said she had to cease exercise because of a chronic condition, *“but I’m happy to get back in it when the flare up dies down again. And I’m very glad there’s always something to go back to”*. It appeared that some of the other participants in the group did not require this motivation because they were intrinsically motivated by the enjoyment of the exercise that they did. For example, one woman in the group had undergone five hip replacements and each time she went through rehabilitation she said her motivation to get better was *“getting back on the ballroom (dancing) floor”.*

*iv) Social norm or friendly comparison of PA:* The final subtheme specifically related to how being a part of the PA program motivated participants to do more than they would alone, or to work at a greater intensity. This was achieved through the social norm of the group or friendly comparison with others in the group. For example, one man commented that “*Another aspect that I thought about then was the activity that I’m doing ... can be quite strenuous, and that is two or three hours, so I’ve got that length of exercise which I probably wouldn’t do if I was doing it by myself”*.

### Social programs

#### Physical activity benefits

There were two mentions and moderate agreement in the social FG discussions that there was also some potential for social programs to provide PA benefits. The sub-theme was coded as iv)‘new opportunities to do PA’. The discussions revealed that socialisation in the groups led participants to learn about PA new opportunities both inside and outside of the LAC. This could be considered both a mechanism and a benefit because if the socialisation is accompanied by a supportive environment it may eventually increase PA levels. For example, one woman said: “Mixing with other people in other different groups; several of them have said to me, “Why don’t you come to our dancing class?” So that’s how I found, you know, um, other avenues into the social fabric of where I am and opportunities”.

### Other subthemes

There were two final program-related subthemes noted in the coding, which appeared to be important for PA adherence in these types of voluntary community programs. Firstly, there was strong agreement from members that availability of the group programs was at very *low cost* compared to other types of PA options available. This was possible because the programs were typically provided by volunteers with a small yearly fee to cover costs. For example, one man commented that, *“the other advantage or the good part about it–with, LAC groups. Is the cost, you know? (All participants agreed)..To, such a minimum cost per year. Because it’s all voluntary work. And so that’s a great advantage really... Because, you know, you just don’t mind spending that small amount of money to stay in a group like that”.* The second program-related sub-theme mentioned by some people that motivated their continued membership were the health benefits they gained from their exercise participation (see next section). See Fig. 2 for a diagram of the linkages between these themes.

#### Physical health benefits of participation in community activity groups

Only PA focus group participants felt that they gained physical benefits from participation in their LAC programs. The benefits fell into two major subthemes: *i) improved physical capacity* and *ii) improved general physical health* (see also Table 3).

*i) Improved physical capacity* was a subtheme describing benefits of program participation on physical ability such as strength and fitness and this translated into an ease of activity of daily living, feeling better and having more energy. For example, one man commented that “*I’m very, very active now and just do everything, that’s so much easier, whether you’re walking or … Whatever you’re doing at home, it’s just so much easier. You just feel so much better*”.

*ii) Improved general physical health* was mentioned by more than half the participants has a key benefit of participating in PA programs. In nearly all cases the benefits were not further elaborated (e.g. “*It’s good mental health as well as physical”).* However, one man did specifically mention that he felt the PA program reduced the disease burden of an existing chronic condition “*I needed the health benefits, joys of being diabetic … dancing three or four times a week to here does help keep, keep you active”*.

There was also reflection among most of the PA program participants that the perceived benefits of participation in their chosen PA program was an additional motivator for continued attendance. For example, one woman said “*And the motivator’s health, continuing good health as long as you can”*.

#### Psychological health benefits of participation in community activity groups

Focus group participants in both groups discussed a variety of psychological benefits relating to involvement in LAC programs. The FG data revealed six main subthemes; *i) adapt to major life events (such as moving-house, retirement or unwell loved ones) ii) cognitive stimulation iii) improved mental health (general) iv) improved life gratitude/life satisfaction/ or QoL v) stress reduction/relaxation vi) enjoyment (enjoying the activities or company of others in the groups or looking forward to the activities).* The number of mentions of each subtheme can be found in Table 3.

*i) Adapt to major life events (such as moving-house, retirement or unwell loved ones):* Many participants described their reason for joining their LAC and the main benefit of membership as being to help them adapt to a major life event. The discussion in both groups was very similar and suggests that both program types offer this benefit to a similar degree. It appears that the reason for this important benefit is that the PA or social programs offer a common activity of interest for connecting socially with others who have similar interests and making new friends at these times of social network flux. One woman in the PA group explained *“I’ve got a new life. Absolutely. [INV: In what way?]. Oh, just meeting people. I’m happier in myself, I was losing all my dance friends because of my partner because with the [Disease] taking over”*. A woman in the social group said *“I do find that most of the enquiries I get for the (for joining a LAC) club are from people who have moved to the area. They’ve moved from interstate to live with their children or be near their children. And they’re looking to start a new social life. And a lot of them do come along to our activities and then gradually get to meet other people”.*

*ii) Cognitive stimulation* was derived predominantly from the activities themselves in ways such as remembering dance steps, learning new skills and mentally challenging games such as Mah-Jong. Though mentioned in both groups, this theme was more prominent in the PA group such as a comment from one of the women: “*dancing’s such a mental thing too. [M1 Yeah]. To remember all the routines and over toes and all the shaping, and there’s just so much to think of all at one time. So it’s good for the brain”*.

In a few cases, the PA group participants summarised the benefits of group membership as being good for their *iii) general mental health*: *“it’s a psychological benefit, isn’t it”*. With further exploration, both groups did identify a number of specific mental health benefits including *iv) improved life gratitude/life satisfaction/ or QoL.* There appeared to be two main ways that quality of life was improved through group participation, this first is through the development of friendships: *“it’s added health and … companionship to my life … it’s a real quality of life improver, definitely”.* The second way is through meeting a variety of people and coming to appreciate the good things in one’s own life such as stated here by one woman in the social group: *“I generally just meet people from different walks of life, and you realize, I suppose, how lucky you are to have what you’ve got. And I think you just meet a, a wider group of the community.”*

*v) Stress reduction/relaxation* was also psychological benefits mentioned in both groups. The activity and the socialisation appeared to offer an escape from daily lives, which aided relaxation. For example, one woman in the social group *said “it’s lovely to just, to be sit down, enjoy everyone’s company and be accepted. And, have a relaxing time, and then go home to reality afterwards”.* In the PA group another woman said *“less stressed and, just much more relaxed. I actually can relax. I definitely feel the benefit. -Hugely”.* Other members of the PA group strongly agreed that participation in the PA activities assisted with stress reduction.

Finally, a key psychological benefit of program participation independent of activity type was *vi) enjoyment*. It was discussed at length in both groups. Enjoyment was derived from enjoying the activities or company of others in the groups or looking forward to the activities. There were a number of reasons given for the enjoyment benefits including socialising and sharing of experiences. For example, one of the women in the social group said: *“If someone’s got a problem with a task, someone’ll say, ‘Does anyone know how to do this?’ and we learn from each other. We have lots of fun, and, um, and, uh, yeah, and we can learn some more crafts. So, yeah, it’s, it’s really good”.*

## Discussion

This mixed methods case study examined the potential effect of becoming a member of an existing community activity group on PA levels and quality of life (QoL), comparing any effects associated with participation in physical activity or social activity programs. Qualitative data obtained through FG interviews assisted with interpretation of quantitative survey data and providing context to the results [65].

The findings of this study suggest that participation in community PA group programs for older adults may assist in maintaining PA levels or motivating older people to do more PA than if they exercised independently. These programs are likely to offer physical health benefits, related to participation in regular PA. In addition, both social and PA community activity programs appear to offer a variety of psychological benefits. The results from the quantitative and qualitative sections of the study are synthesised in the discussion below.

### Physical activity (PA)

The synthesis of survey and FG findings suggest that the primary benefit of the PA programs was the maintenance of PA levels. The survey results demonstrated that PA levels of the PA program participants remained stable over one-year. In contrast, the PA levels in the social group appeared to decline; which likely reflects age-related declines in PA [14–17]. The high degree of variance in PA scores and small sample size in the survey made interpretation challenging but FG study results also suggested that PA programs assist with PA maintenance. The potential benefit of groups for promoting PA for older adults is not a new concept. Various aspects of group dynamics have been successfully utilised in to significantly increase PA levels by between 1.5 [48, 49] and 2 days per week [51] in past behavioural intervention studies, which focused on promoting PA behaviour outside of the programs. In these studies, using group dynamics to motivate PA behaviour appeared to be particularly beneficial for people aged between 60 and 75 years [51] and for those who had less social support outside the program [48, 49]. Each of the studies found that the least active participants increased their PA levels the most [48, 49, 51]. The present study found that PA was only maintained but the results above suggest that this is likely to be because the cohort was already active, with 80% already meeting the PA guidelines [8]. Group exercise training for older adults [50] has also been successful in significantly increasing PA levels in older adults but the benefits were limited to the intervention period (8 weeks) and not maintained past 6 months. Whilst not all older adults will want to attend group PA, there are clearly people who benefit from it and as demonstrated above, short group PA programs are not typically effective in maintaining PA long term [50].

The main novel findings of this study were the suggestions of *why* low-cost socially- focused community group programs may assist in maintaining PA levels in older adults, which emerged from the mixed methods design of this study, which is different to what was used in the studies above. There was consistent agreement that the group PA environment, social interaction, as well as perceived health and wellbeing benefits from group participation, assisted the enjoyment and motivation to attend weekly PA programs (see Fig. 2). This is consistent with previous qualitative research, for example a recent study reported that inactive older men who undertook team sport activity were more likely to continue PA than men in individual sport activity [70]. In line with the present study, they felt the team sport environment improved their motivation for attending through enjoyment of socialisation and relatedness with others [70]. Similarly, a study of older women reported that the social connections and support in the group were a reason to ‘stick with’ their exercise classes [68]. Furthermore, a recent systematic review highlighted the importance of a social setting to older adult PA participation, with peer interaction being a key factor facilitating enjoyment and motivation for PA in 64% of studies reviewed [71].

Whilst the survey study findings suggest that joining a community group PA program maintains but may not increase PA levels in those who are already physically active, the FG study findings suggest that the PA activity groups encourage some of these people to do more PA or at a greater intensity than if they exercised alone. There was agreement by participants in the FGs that being part of the group provided a ‘social norm’ for PA which was motivational. This social norm for positive behaviour is an established mechanism linking cohesive social groups and good health [72]. Like any behavioural strategy however, the impact will depend on each individual’s PA behavioural correlates, which vary between individuals [71]. Thus, the mixed result observed in our study is not surprising.

There are several potential contributors to the lack of observed change in PA over time in the survey study. The first is the active cohort which was mentioned above. Secondly, a small sample size combined with use of a self—report PA scale, which are known to be less accurate than objective measures such as accelerometers, would have contributed to the variability of the results [69]. For example, an experimental design study with older adults investigating interpersonal strategies for increasing PA levels in sedentary older adults found that they were effective in increasing the PA levels of sedentary adults compared to no intervention when measured by objectively measured PA levels but not by self-reported PA scale [19]. Thus, it is possible that the self-report method used for PA reporting in the current study, was not sensitive enough for the small numbers in this sample or the small changes that are likely to have occurred in the active cohort.

One interesting and unexpected FG finding from this study was that some people in the social group gained PA benefits from joining their LAC through finding out about other PA opportunities from fellow participants. This is a particularly useful benefit of offering both social and PA activities at one organisation. The novel benefit of being part of a large diverse club offering both PA and non-PA type activities, that would not be possible in single-activity clubs, has not been previously identified in other research literature.

### Physical health-related quality of life

Similar to previous research in the UK [73], in this study, physical HR QoL was significantly higher at baseline in the PA group than the social group. It is likely that people who are have better physical health through being physically active throughout their lives, are likely to join a PA program and people with poorer health may prefer social groups [74].

The synthesis of the FG and survey results suggest that physical health benefits are gained through the PA programs but not necessarily through social programs. The impact of becoming a member on physical HR QoL did not differ over time between groups (i.e. no significant group by time interaction). A significant interaction effect would have confirmed this finding but post-hoc analysis did suggest some group differences may have been present. The lack of strong statistical evidence relating physical HR QoL to group activity is likely to be due to type II error from a small sample and biases of self-report surveys. However, the FG discussions suggested that participants perceived that the PA they did in their LAC program offered them significant physical health and physical capacity benefits; known to be associated with regular PA [6–8]. This contrast in results suggest that larger studies are warranted in future to confirm any association.

In addition to the small sample size limiting the likelihood of observing significant differences between groups, it may also be that some physical benefits can be derived from either kind of program or that membership of such programs increasing time out of home and this maintains physical function. Other studies have found a significant protective effect of being a member of either hobby groups (social groups) or PA groups, on the onset of disability and against declines in self-rated physical health in older adults when following up over a four-year period [35] and that better physical function is signifcantly related to more time out of home in older adults [75].

### Mental health-related quality of life

The MCS scores in the survey study did not change significantly over the one-year study in either program group or between groups. However, FG participants felt that both social and PA programs have the potential to provide multiple mental health benefits. This discrepancy may have been because the survey participants already reported good mental health-related quality of life (HR QoL) at baseline, making it unlikely to observe further changes in the scale used (a ceiling effect). Another option suggested by the FGs is that participation in these kinds of group programs may maintain mental HR QoL or subjective wellbeing, which may otherwise decline. This would be in line with past research, which has consistently found that either social activity or physical activity participation may protect against declining mental health (including depression) and maintaining good quality of life in older adults [73, 76, 77]. Some of the benefits discussed by participants of the FGs in this study included adaptation to major life events, activity based cognitive stimulation, improved QoL and stress reduction, activity enjoyment, and socialisation. These benefits have been noted as being valuable for older adults and likely to be associated with better wellbeing [78, 79] and an ability to age ‘Actively’ or ‘Successfully’ [5].

This study adds to the literature regarding the benefits for physical activity, physical and mental HR QoL through participation in multi-activity community groups for older adults. It suggests that group PA programs in such organisations may assist with maintenance of and possibly increasing PA levels of older adults, especially when they require new avenues for social participation (e.g. after moving or retiring). It would be worthwhile investigating if the same associations would be observed in participants with low physical activity levels or who are initially inactive. In relation to physical health-related QoL, the LAC PA programs appear to offer benefits in line with undertaking regular PA.

## Strengths and limitations of the study

This mixed-method case study combined a longitudinal quantitative study with qualitative FG discussions. The strength of this approach was that observations relating to the quantitative variables (PA, and physical and mental HR QoL) could be explained, contextualised or expanded upon with the qualitative data. This was particularly beneficial in this study where the quantitative data suffered from limited power due to a small sample size, caused by recruitment difficulties common to observational studies. These were fewer than expected new members joining LACVI during the recruitment period and a lower than anticipated rate of promotion of the study by some of the LAC clubs, resulting in low participant numbers, despite an extended recruitment period of 2 years.

Embedding the research in an existing community organisation was a major strength of this study. The real-life setting allowed us to evaluate whether existing programs can be effective with the types of people who naturally choose them. In our case, this was particularly relevant because people with different interests may choose either PA or social programs, but the effect of individual preferences on results would have been lost in a more controlled or randomised setting [80]. The real life setting also made it low cost and made drop-out very low, as people were already choosing to join the club. However, there are also clear limitations of a self-selecting participant group. People who chose to take part in the study were already quite active and reported good health-related QoL and were not isolated. This made it more difficult to evaluate whether these organisations may increase PA and QoL in inactive older adults or those who are harder to reach and not naturally inclined to join a community organisation. It also means that the findings can only really be generalised to club seekers of similar organisations [81]. Future studies expanding on this work would aim to explore how to encourage people who are less active and not club seekers to these programs. Self-selection also meant that more women than men took part.

As mentioned earlier in the discussion, use of self-report surveys is a limitation of this study, especially in relation to PA estimations, where the standard errors and interquartile range were large in this study. It is well known that self-report surveys may suffer from recall bias due to a reliance on memory and subjective nature of interpretation of the questions. This is especially the case in older people [82]. Objective markers of PA such as accelerometers are more accurate in assessing PA levels, especially in smaller cohorts. Unfortunately, resource and practical constraints made use of accelerometers impossible in this study.

Whilst the study population was described as ‘older adults’, the age range of participants in this study was wide, being between 45 and 80 years because LAC accepts members from age 45 to encourage people to consider group membership before retirement. There is a wide variety in what is generally considered as the age cut-off for ‘older adults’. Research in sports settings where physical health limitations may limit ability may define older to be 50 years [73]. Whereas, the generally agreed WHO definition is 65 years of age [5]. The mean age of participants included in the study sits around the accepted definition of older adults by the UN, which is 60 [1]. Age was included as a covariate in the analysis.

## Directions for future research

The results of this small study were promising and suggest that future larger studies would be warranted to evaluate existing multi-activity community groups for older adults on a larger scale. The population group in this study was already quite physically active, therefore it would be beneficial to assess if community organisations for older adults may also assist inactive older adults. This would probably require a specific strategy for recruitment of inactive participants to the organisations. The case study nature of the research made generalisability difficult, so it would be beneficial to expand the qualitative and quantitative studies to include other types of community organisations to investigate if this finding is indeed generalizable outside LACVI. We would recommend use of objective PA measurements (e.g. accelerometers) if possible to accurately collect PA data. Sex-stratified FGs would also be beneficial to investigate whether there are any differences that exist between men and women.

Given the novel finding that social relationships developed in social programs may encourage previously inactive people to try new things such as physical activities, there may also be scope for interventions to gently introduce opportunities to do PA within the same organisation. One option may be for members of PA programs to join some social programs and gently promote another, low impact enjoyable activity such as social walking or dancing available through the same organisation.

## Conclusion

With an ageing population it is important to investigate ways to enable older adults to age successfully to ensure optimal QoL. Community activity programs offering group physical activities may maintain PA levels in older adults. It appears that either social or PA groups may also offer benefits to maintain good perceived physical health and mental health-related QoL in older adults through socialisation and enjoyment. In conclusion, ageing policy and strategies should consider community activity groups for older adults as potential low-cost and sustainable options for promoting PA and QoL for older adults.

## Data Availability

The datasets generated and/or analysed during the current study are not publicly available due the ethics approval for this study not allowing open access to the individual participant data but are available from the corresponding author on reasonable request.

## Abbreviations

AR1: First-order autoregressive correlation structure
FG: Focus group
HR QoL: Health-related quality of life
IQR: Interquartile range
LAC: Life Activities Club
LACVI: Life Activities Clubs Victoria
LMM: Linear mixed model
M: Mean
MCS: Mental health component of SF-12
PA: Physical activity
PCS: Physical health component of SF-12
QoL: Quality of life
SE: Standard error
WHO: World Health Organisation
